# Two cases of methyl alcohol intoxication by sub-chronic inhalation and dermal exposure during aluminum CNC cutting in a small-sized subcontracted factory

**DOI:** 10.1186/s40557-016-0153-9

**Published:** 2016-11-15

**Authors:** Jia Ryu, Key Hwan Lim, Dong-Ryeol Ryu, Hyang Woon Lee, Ji Young Yun, Seoung-Wook Kim, Ji-Hoon Kim, Kyunghee Jung-choi, Hyunjoo Kim

**Affiliations:** 1 Department of Occupational and Environmental Medicine, Ewha Womans University College of Medicine, Seoul, South Korea; 2 Department of Ophthalmology, Ewha Womans University College of Medicine, Seoul, South Korea; 3 Department of Internal Medicine, Ewha Womans University College of Medicine, Seoul, South Korea; 4 Department of Neurology, Ewha Womans University College of Medicine, Seoul, South Korea; 5 Department of Emergency Medicine, Catholic University College of Medicine, Seoul, South Korea; 6 Department of Occupational and Environmental Medicine, Ewha Womans University Mokdong Hospital, 911-1, Mok-6-dong, Yangchun-gu, Seoul, 158-710 South Korea

**Keywords:** Methyl alcohol intoxication, Optic neuropathy, Metabolic acidosis, Vulnerable workers

## Abstract

**Background:**

Methyl alcohol poisoning has been mainly reported in community. Two cases of methyl alcohol poisoning occurred in a small-sized subcontracted factory which manufactured smartphone parts in Korea.

**Case presentation:**

One young female patient presented with dyspnea and visual disturbance. Another young male patient presented with visual disturbance and myalgia. They treated with sodium bicarbonate infusion and hemodialysis for metabolic acidosis. In addition, he received ethyl alcohol per oral treatment. Her and his urinary methyl alcohol concentration was detected as 7.632 mg/L, 46.8 mg/L, respectively, although they were treated hemodialysis. Results of the working environment measurement showed that the concentration of methyl alcohol (1030.1–2220.5 ppm) in the air exceeded the time weighted average (200 ppm). They were diagnosed with optic neuropathy due to methyl alcohol poisoning and still have visual impairment.

**Conclusions:**

Workers who hired as dispatched employees in a small-sized subcontracted factory were exposed to high concentrations of methyl alcohol. The workplace had poor ventilation system. In addition, workers did not wear proper personal protect equipment. Working environment measurement and annual chekups for workers were not performed. They were in a blind spot to occupational safety and health. More attention is needed to protect vulnerable workers’ health.

## Background

Methyl alcohol known as wood alcohol is a colorless and volatile liquid. Methyl alcohol smells of drinking alcohol. Methyl alcohol is used as a production of formaldehyde, paints, cement, and inks. Absorbed methyl alcohol from oral, dermal, and inhalation is easily distributed in the body [1]. Symptoms of methyl alcohol intoxication are characterized by visual impairment and metabolic acidosis. Methyl alcohol intoxication without proper treatment is responsible for high mortality [2].

Two patients who had visual disturbance that are often represented by the symptom of methyl alcohol poisoning were occurred in a factory which manufactures smartphone parts. Smartphones are commonly used in South Korea as well as all over the world. Many factories which manufacture smartphone parts were established to meet the increased demands for use a smartphones. These factories mostly hire workers as the dispatched workers employed by temporary work agencies. Electrical and electronic industry ranks fourth to hire the subcontractor employees in the manufacturing business, subsequently shipping, steel, machine industry [3]. Workers in a subcontracted firm are more likely to have a risk which occurred from physical and chemical factors than workers at parent firms [3]. The patients worked as the dispatched workers who were more vulnerable to occupational health than regular employees. We report these two cases of methyl alcohol poisoning in electronic industry.

## Case presentation I

### Patient

Twenty-eight years old, Female.

### Chief complaint

At 15:40 on January 16, 2016, the patient visited emergency department and was presented with dyspnea accompanied by visual disturbance.

### Present illness

She had a 3 months history of common cold like symptoms including cough, headache and fatigue. Her Symptoms did not differed depending on the times, day-and-night and working or not. On January 15, she worked as night shift from 21:00 to 9:00. She felt nausea and vomited once on duty. She visited a local hospital and received a blood test during on duty at 22:00. However she heard that she doesn’t have any clinical problems. She came back to work and continued to work. In the morning of January 16, she had dimmed vision shortly before she was dismissed from the work. However she thought that the symptom was caused by fatigue and just went to home. She went to sleep when she arrived home. When she waked up after 5 h, she had difficulty to breathe and chest tightness. Dyspnea was aggravated gradually. At the same time, the appearance of black curtain over the upper and lower side of visual field occurred.

### Past medical history

No specific past medical history.

### Social history

No specific hobby history, no specific drug history, the married state.

### Smoking history

One pack × 9 years = 9 pack-years.

### Alcohol history

Soju, 2 bottles per week.

### Occupational history

According to the interview with her and her husband, she had worked in a semi-conductor factory which was located in Bucheon for 8 years. She quitted the job and rested for 6 months. She started working at a small factory which manufactured cell phone parts in Bucheon since September, 2015. She worked to find defected goods placing her own face toward the machine closely. She used air-gun to remove remaining solvent on aluminum plate without proper personal protectors like mask and gloves. She has worked as a shiftwork employee who inspects the finished products.

### Family history

No specific family history.

### Physical examination

In emergency room, initial vital signs were as follows: blood pressure 144/80 mmHg, pulse 78 beats/min, respiration 28 breaths/min, body temperature 36.1 °C, and saturation of O2 100%. Upon auscultation, clear breathing sound without rale and wheezing was heard in both lung fields. Regular heart beat without murmur was heard.

### Diagnostic assessment

Her electrocardiogram indicated normal sinus rhythm. She received arterial blood gas analysis (ABGA) and pulmonary embolism computed tomography (CT) to evaluate the cause of dyspnea. The initial ABGA (pH 7.131, pCO2 10.3, pO2 132, Base excess -26, Bicarbonate 3.5) and serum electrolyte results (Na^+^ 138, K^+^ 4.7, Cl^−^ 106, tCO_2_ 6 mEq/L, anion gap 26) supported that she had increased anion-gap metabolic acidosis with respiratory compensation. In addition, pulmonary embolism CT was negative.

### Interventions

She was immediately treated with sodium bicarbonate. Despite the continuous bicarbonate infusion, metabolic acidosis as well as her physical status was not improved. Rather, her mental status worsened to drowsiness with decreased respiration. Emergency intubation was performed immediately and she was put on a ventilator (Pressure regulated volume control (PRVC) mode: tidal volume 380 ml, respiration 8 breaths/min, fraction of inspired O2 40%, positive end-expiratory pressure 5cmH2O).

**Fig. 1:**
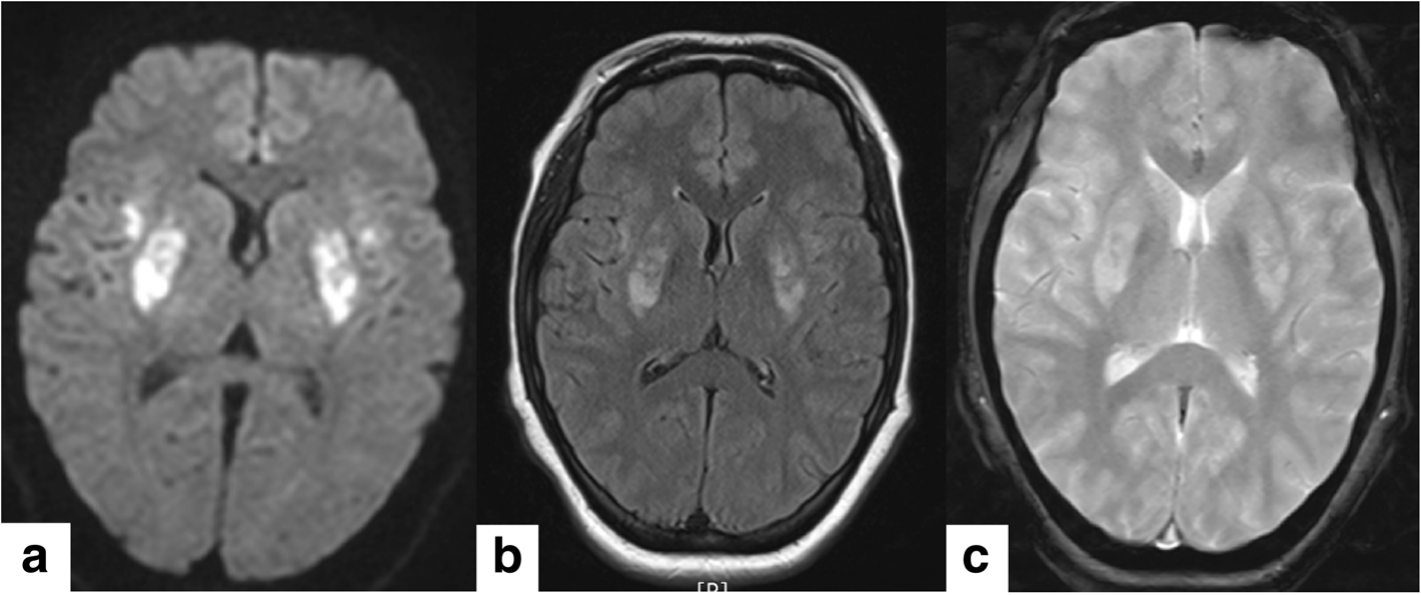
Brain magnetic resonance image (MRI) showed abnormal signal intensity in the bilateral putamen and anterior insular cortices. **a** diffuse restriction in the bilateral putamen and anterior insular cortices in diffusion weighted image, **b** high signal intensities in T2-weighted image on the same region, **c**: focal hemorrhage was not detected in susceptibility weighted image

Brain magnetic resonance image (MRI) was taken to confirm a problem about respiratory control center. MRI results did not indicate respiratory control center problem. However it showed diffuse restriction in bilateral putamina, bilateral anterior insular cortices, and bilateral medial frontal cortices. It needed to rule out subtle diffusion restriction in bilateral precentral cortices. It also showed peripheral rim enhancement in bilateral putaminal lesions (Fig. 1a, b, c). Thereafter, she was admitted in intensive care unit (ICU) under the nephrology department.

### Follow-up and outcomes

On January 17, she still had drowsy mental status and was assisted by mechanical ventilator to breath. Arterial blood gas analysis identified that she still had increased anion-gap metabolic acidosis (pH 7.220, pCO2 16.0, pO2 149, Base excess -20.2, Bicarbonate 6.3, Na^+^ 143, K^+^ 3.4, Cl^−^ 113, tCO_2_ 6 mEq/L, anion gap 24). On January 17–18, she received hemodialysis treatment once a day. This hemodialysis allowed her to recover from drowsy mental status as well as metabolic acidosis (pH 7.484, pCO2 32.5, pO2 93.8, Base excess 1.6, Bicarbonate 24.1).

On January 18, the nephrologist requested a consultation with a specialist of occupational and environmental medicine, because he heard from her family that the patient used to smell of liquor after work. The specialist of occupational and environmental medicine interviewed her about her occupational history including information of working process. She did not know that what kinds of materials she used in the factory. However, the specialist of occupational and environmental medicine suspected that this case was a work-related disease and recommended that her urinary and blood samples need to be collected to analyze exposure materials. Her blood and urinary samples were collected on January 19.

On January 19, 2016, she was transferred to general ward. She complained that she had blurred vision and failure to distinguish objects and person. Moreover, she showed binocular pupil dilatation. On January 20, 2016, she was transferred to ophthalmologist who performs a special ophthalmologic test following visual acuity test, visual field test, color vision test, fundus examination and optical coherence tomography (OCT). Her visual acuity was as follows: right (finger count 20 cm), left (finger count 30 cm). Both pupils were slightly dilated with light reflex. Isihara color vision test results were as follows: right (0/24), left (2/24). Moreover, OCT indicated that both optic nerves were edematous. She was diagnosed with toxic optic neuropathy by ophthalmologist and treated with steroid.

On January 21, 2016, the neurologist had an exam about her brain function and confirmed that she spoke abnormally quiet and acted too slow. Specific tests including mini mental state examination (MMSE), electroencephalography (EEG) and nerve conduction velocity (NCV) were taken to confirm neurologic problems. Her MMSE score was in the normal range (28/30). EEG showed diffuse symmetric theta to delta slow activities in the bilateral hemispheres, suggesting moderate to severe diffuse cerebral dysfunction, which is compatible with toxic or metabolic encephalopathy. NCV revealed no specific abnormality. She was diagnosed with toxic encephalopathy by neurologist and treated with choline alfoscerate. On January 25, 2016, she discharged to home with near complete nephrologic recovery. However other conditions such as ophthalmologic and neurologic dysfunction were not fully recovered until March 31, 2016.

Her urinary sample was analyzed to detect a concentration of methyl alcohol after the inspection of her factory confirmed that she had been exposed to 99.9% methyl alcohol in the workplace. Her urinary methyl alcohol concentration was detected as 7.632 mg/L (Biological Exposure Indices (BEI) is 15 mg/L of methyl alcohol in urine at end of shift) although 3 days had passed since she stopped work and she was treated hemodialysis twice.

## Case presentation II

### Patient information

Twenty-seven years old, Male.

### Chief complaint

At 01:10 on January 22, 2016, the patient visited emergency department and was presented with blurred vision and ophthalmalgia accompanied by myalgia.

### Present illness

He often had nausea since he started to work in the factory in September, 2015. However, this symptom subsided by taking some foods. Although he felt dizziness, he worked as night shift from 21:00 to 9:00 on January 20. In the morning of January 21, he had blurred vision shortly before he got off work. He also thought that the symptom was caused by his own fatigue. He went to sleep in his home and waked up at the evening of January 21. At that time, he felt blurred vision as well as both eye ball pain. Moreover, he had weakness accompanied by mild myalgia. He called his manager and said that he could not attend to work today. He took a cold medicine and went to sleep again. When he waked up at midnight of January 22, he had difficulty to distinguish objects. Until then, he could not hear that his coworker who had had similar symptoms was admitted to hospital.

### Past medical history

No specific past medical history.

### Social history

No specific hobby history, no specific drug history, the unmarried state.

### Smoking history

0.5 pack × 8 years = 4 pack-years.

### Alcohol history

Beer, 2 bottles per week.

### Occupational history

He was a migrant worker from China. He could speak Korean and communicate with other workers. He did not work any kinds of specific work in china. After he immigrated to South Korea, he worked lathe operators during 3 months. He quitted that job and started working at the same factory where the patient 1 has worked since September, 2015. He has worked as a shiftwork employee who manufactures the mobile phone parts by using the CNC machines. He used air-gun to remove remaining “alcohol” on aluminum plate without proper personal protectors like her. Sometimes, the solvent was spattered to his eyes. He rubbed his eyes to remove the solvent using his hands which were wetted with the solvent. He also did not know that what kinds of the solvent, “alcohol” he has been exposed in the factory.

### Family history

No specific family history.

### Physical examination

In emergency room, initial vital signs were as follows: blood pressure 130/80 mmHg, pulse 112 beats/min, respiration 20 breaths/min, body temperature 36.1 °C. His both eyes indicated mydriasis.

### Diagnostic assessment

He was transferred to ophthalmologist who performed a special ophthalmologic test following visual acuity test, pupil examination, fundus examination. His visual acuity was as follows: right (hand motion perception), left (finger count 10 cm). Both pupils were dilated without light reflex (right 5.5 mm, left 6.4 mm). In addition, blurry optic disc margin with swollen optic disc was showed by funduscopy.

### Interventions

At afternoon on January 22, he received MRI orbit to find the cause of decreased visual acuity and spinal tapping to exclude the neurologic problems. MRI orbit showed bilateral tram track sign like T2 signal hyperinstensities with enhancement along the both retrobulba intraorbital segment of both optic nerve. After that, he was admitted in general ward under the ophthalmology department. The ophthalmologist diagnosed him with both optic neuritis and treated with steroid.

### Follow-up and outcomes

On January 23, he and his guardian spoke to the ophthalmologist that his coworker having visual disturbance was admitted in other hospital and diagnosed with methyl alcohol induced toxic optic neuropathy. The ophthalmologist requested a re-consultation with a specialist of emergency medicine. On January 24, he was transferred to ICU under the emergency medicine department. He received ABGA and was started hemodialysis treatment, leucovorin infusion per intravenous and 40% ethanol (Vodka) per oral treatment (loading dose 1.8 ml/kg, maintenance 0.5 ml/kg/h). The initial ABGA (pH 7.356, pCO2 26.8, pO2 121.8, Base excess -9.0, Bicarbonate 14.7) and serum electrolyte results (Na^+^ 139, K^+^ 3.5, Cl^−^ 105, tCO_2_ 8 mEq/L, anion gap 19) were confirmed that he had increased anion gap metabolic acidosis with respiratory compensation. On January 25, the follow up ABGA (pH 7.473, pCO2 32.1, pO2 99.2, Base excess 0.0, Bicarbonate 23.0) showed that he recovered from metabolic acidosis. On January 24–26, he was treated with hemodialysis once a day. On January 24–27, he was treated per oral ethanol treatment, too. On January 25, the neurologist had an exam about him to find neurologic deficits. Additionally, brain MRI and EEG were taken, which did not show any specific abnormality.

On January 26, patient’s urinary and blood samples were collected by recommendation of the specialist of occupational and environmental medicine in the hospital where his coworker was admitted. His urinary methyl alcohol concentration was 46.8 mg/L. On January 27, he was transferred to general ward. On February 1, he was finally diagnosed with bilateral optic neuropathy due to methyl alcohol intoxication. He discharged to home with ophthalmologic dysfunction, although he recovered other conditions such as metabolic acidosis and myalgia.

## Exposure assessment

The specialist of occupational and environmental medicine shared the information of suspected intoxication case with the authorities concerned on January 19. The Bucheon Regional Ministry of Employment and Labor recognized the case and measured air-concentration of methanol on January 22. A labor supervisor in Bucheon Regional Ministry of Employment and Labor inspected the factory on January 22. The supervisor conducted working environment measurement to assess exposure level of workers. Total five samples were obtained from personal sampler. The factory used 99.9% methyl alcohol. These air samples which were randomly collected from five spots from 18:30 to 23:30, during 5 h, on January 22 were detected with methyl alcohol in the range of 1030.1 ppm to 2220.5 ppm (8-h Time Weighted Average, TWA 200 ppm; Short Term Exposure Limits, STEL 250 ppm). Gas chromatography (GC) was performed on an Agilent 7890 series gas chromatograph (Agilent Technologies) with a flame ionization detector. Specific analysis conditions were as follows: desorption H2O, injection volume 1ul, injection temperature 240 °C, nitrogen carrier gas, capillary column (HP-5 : 30 m × 0.32 mm × 0.50 μm film thickness).

In the morning on January 25, first patient’s husband informed to the occupational physician that the second case were hospitalized due to similar symptom. The offices ordered to stop the all process of work toward the factory where she had worked. In the afternoon on January 25, the labor supervisor accompanied the specialist and a resident of occupational and environmental medicine to the factory. This factory was established on May, 2, 2011. About 30 people were hired as day and night, double shift (from 9:00 to 21:00 and 21:00 to 9:00). Overtime work did not to exist. Most workers including two patient2 dealt with computer numerical control (CNC) machine to produce a cell phone parts. Two workers including patient1 were responsible for inspection a finished products. The factory had not conducted a working environmental measurement. The manager said that other analogous cases like her had not occurred since established the factory.

**Fig. 2:**
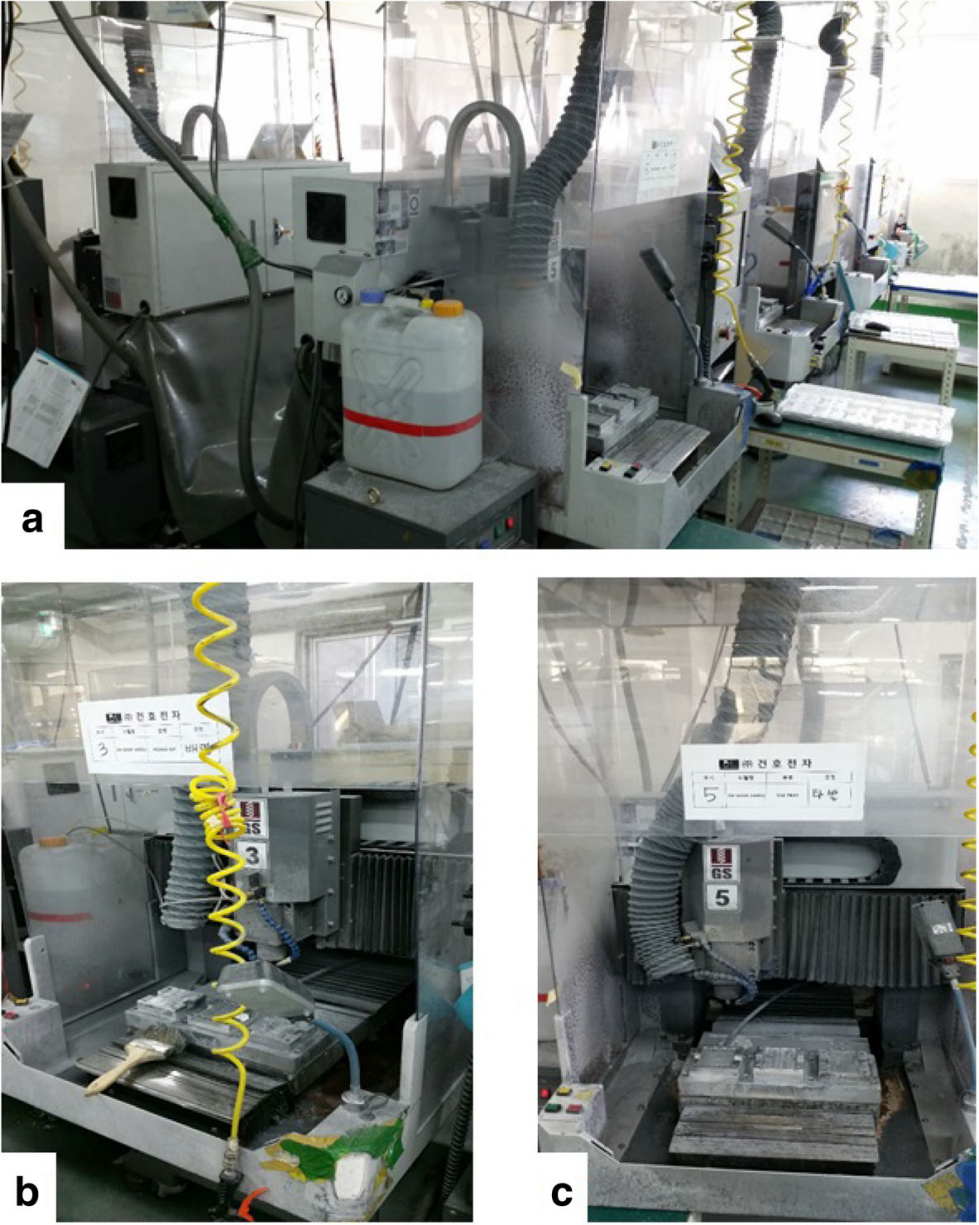
**a** CNC machines were arranged in a row. About 30 machines operated on duty. A plastic drum which did not have any hazard marker contained methyl alcohol. **b** and **c** CNC machines were always open. *Gray* rubber hose was ventilating system. However, these did not operate properly

CNC cutting machine was used to manufacture cell phone parts made by aluminum plate (Fig. 2). This machine produced repeated identical products by spraying a methyl alcohol continuously to cool down the aluminum plate. CNC cutting machine was not closed type but open type. During this step, methyl alcohol vapor and aluminum dust were scattered to the air. Local exhaust ventilation system was equipped, but did not operate properly.

Two patient’s collected urinary samples were considered the temporary health checkups for workers. GC was performed on an Agilent 7890 series gas chromatograph (Agilent Technologies) with a flame ionization detector. As noted above, her and his urinary methyl alcohol concentration was 7.632 mg/L, 46.8 mg/L, respectively.

## Discussion

These two cases showed subacute methyl alcohol poisoning occurring workers who had worked without any information on the hazard and proper protective system in a factory manufacturing smartphone parts. Methyl alcohol poisoning has been mainly reported in community as accidents or crimes by oral ingestion [4] or transdermal absorption [5, 6]. Two industrial cases of methyl alcohol intoxication were found in a poorly ventilated laboratory chronically [7] and a methyl alcohol production factory with acute exposure [8]. Smartphones have become common in used all over the world. The increased demand for the smartphones has triggered an establishment of factory in which smartphone parts are produced. Given this global situation, these two cases are sentinel cases that alarm all related workers, employers, researchers, activists, and government officers.

According to the practice guideline on the treatment of methanol poisoning of the American Academy of Clinical Toxicology (AACT), major clinical symptoms of methyl alcohol intoxication are presented with central nervous system, eyes, and gastrointestinal tract. Severe metabolic acidosis with high anion gaps may be used as important evidence to confirm a methyl alcohol poisoning. Moreover, the common findings of the imaging studies in methyl alcohol poisoning are bilateral changes of the putamen, cerebral edema, and subcortical white matter change [9]. The diagnosis of methyl alcohol intoxication in our patients was made based on the history of the excessive exposure to methyl alcohol, and the specific symptoms such as visual disturbance, nausea, and vomiting, and metabolic acidosis with high anion gap. Moreover, through history taking to our patients, we confirmed that our patients did not take any methyl alcohol. Her brain MRI was shown common findings in methyl alcohol intoxication. The patients’ urinary samples were confirmed that the patients had methyl alcohol in their body.

Methyl alcohol is rapidly absorbed from various routes of exposure like skin, respiratory system, and gastrointestinal tract. In the liver, methyl alcohol is oxidized to formaldehyde by alcohol dehydrogenase and to formic acid by formaldehyde dehydrogenase. Formic acid is toxic metabolite that causes the metabolic acidosis as well as the optic nerve toxicity. Thus, susceptibility to methyl alcohol toxicity depends on the rate of formic acid clearance. Formic acid is excreted as formic acid in the urine or transformed to carbon dioxide and then eliminated by exhaling [2]. About 1.5% of absorbed methyl alcohol from ingestion or inhalation is excreted in the urine as methyl alcohol. However, urinary methyl alcohol is more sensitive than urinary formic acid to detect the exposure to methyl alcohol vapor [10, 11]. According to the American Conference of Governmental Industrial Hygienists (ACGIH) guideline as well as the Korea Occupational Safety and Health Agency (KOSHA) guideline, biological monitoring of methyl alcohol exposure needs to be performed by measuring the urinary methyl alcohol [1]. Therefore, urinary samples of workers were collected and analyzed to monitor the concentration of methyl alcohol in urine.

The target organ of methyl alcohol is the retina. Formic acid is more selectively accumulated in eyes because the oxidation of formic acid in the eye is slower than in other organs. Formic acid is found in the vitreous humor and the retina. Formic acid inhibits cytochrome oxidase c, which leads to the impairment of ATP production, followed by the depletion of energy in the cell. These changes can appear retinal toxicity of methyl alcohol [12]. Our two patients had decrease visual acuity and edematous optic disc. In addition, the patient 1 had difficulty to distinguish colors. All these symptoms were caused by the changes of nerve cells in retina. Another main symptom of methyl alcohol intoxication is metabolic acidosis. Increased hydrogen ion occurring from excessive production of acids such as formic acid leads to metabolic acidosis. It stimulates both central and peripheral chemoreceptors. Respiratory compensation leads to increased minute ventilation which causes increased tidal volume, followed by the rising respiratory rate. This pattern of respiration may be a cause of dyspnea [13, 14]. Through the exposure to methyl alcohol, two patients had excessive concentration of acid from methyl alcohol metabolism in their body, resulting in metabolic acidosis. Accumulated methyl alcohol in their body continuously converted to formic acid, which can aggravate metabolic acidosis. The two patients recovered from metabolic acidosis only after applying a hemodialysis treatment which removed methyl alcohol as well as formic acid in their body.

Although ingestion accounts for dominant proportion of the methyl alcohol poisoning, inhalation of high level of methyl alcohol and percutaneous absorption of methyl alcohol liquids are similar to toxicity of methyl alcohol from ingestion. Ocular toxicity like blurring, narrowing of visual field, and loss of vision also have been reported in occupational situations show that methyl alcohol concentration in air about 1200 ppm or more [15]. Moreover, these days some studies have been reported that critical clinical manifestations following inhalation of methyl alcohol [8, 16–18]. Two different routes of exposure like inhalation and ingestion have some differences. Ingestion permits larger dose of methyl alcohol than inhalation. Inhalation reaches quickly the peak level of methyl alcohol by pharmacokinetic theory [19].

The patients who are suspected to have methyl alcohol poisoning need to be treated to correct metabolic acidosis [20, 21] and inhibit metabolism of methyl alcohol by using fomepizole or ethyl alcohol. In addition, folate treatments help excrete formic acid out of body [2]. Although patients receive proper management, patients who have persistent metabolic acidosis or visual symptoms are treated with hemodialysis. These steps need not to be waited until the confirmation of methyl alcohol concentration [9]. In the first case, she was admitted to ICU and treated with sodium bicarbonate infusion to correct metabolic acidosis. After receiving the treatment, she still had uncorrected severe acidosis (pH <7.3) and treated with hemodialysis. However, she could not receive fomepizole, ethyl alcohol, or folate treatment. It took about two days until the doctors were suspicious of her disease as an occupational intoxication and additional four days until they knew that her exposure material was methyl alcohol. This was because methyl alcohol intoxication has been rare in South Korea and the patient did not know what she has exposed in the workplace. In the second case, he was admitted to ICU and treated with ethyl alcohol ingestion, leucovorin administration, and hemodialysis. Because his doctor heard that patient’s symptoms were related to methyl alcohol, he could receive treatment suited for methyl alcohol poisoning.

Ethyl alcohol or fomepizole can inhibit formation of toxic metabolites such as formic acid by blocking the metabolic pathway of methyl alcohol [2, 22]. Ethyl alcohol is a competitive substrate for alcohol dehydrogenase and fomepizole is a competitive inhibitor of alcohol dehydrogenase. Fomepizole is approved as the antidote to treat a methyl alcohol intoxication in the United States in 2000. This drug has no contraindications and no side effects except allergic reaction. The AACT present that fomepizole is a first-line agent in methyl alcohol intoxication. Moreover, many experts recommend that immediate fomepizole therapy needs to be given to patients who are suspected to have methyl alcohol poisoning [22]. However, only 14 regional emergency medical centers have fomepizole in South Korea [23] that could make barriers for other hospitals to use it appropriately.

Most laboratory findings including pH, bicarbonate level, anion gap, osmolar gap, and methyl alcohol level are related to final visual acuity. Patients who have pH greater than 7.2 tend to show transient visual impairment [24]. The visual loss due to methyl alcohol intoxication can be either reversible or permanent. Our both patients were not fully recovered from visual disturbance yet. In addition, patients of methyl alcohol intoxication may contract with parkinsonism whose symptoms are rigidity, bradykinesia, mild tremor, and dementia. A young patient with long-term exposure to methyl alcohol in laboratory develop parkinsonism as delayed toxic effect [7, 9]. Both visual impairment and neurologic impairment are likely to continue or worsen [4]. Our patients have only visual impairment without neurologic deficit. However, there is possibility that new symptoms appear as delayed symptoms. Therefore, the closed follow up is required for patients to observe future complications.

Several important systemic problems were involved in the occurrence of these cases. First, methyl alcohol is cheaper than ethyl alcohol. The factory used methyl alcohol which has replaced the ethyl alcohol. Employers as well as employees knew nothing of the health effects of methyl alcohol. Second, the industrial safety and health acts including education about hazard materials for workers, operation of proper ventilation system, providing for personal protection equipment, regular working environment measurement, and regular checkups for workers are applicable in the factory. However, employers were not aware of the facts that the acts were applicable in their factory and did not do anything. Third, most workers were dispatched workers employed by temporary work agencies who had the mean length of service of their company was three months. For this reason, personal management was improperly executed. In addition, prime company was not interested in management of the factory and subcontractor employees’ health.

Some strategy needs to prevent recurrence of methyl alcohol poisoning in workplaces. First, for similar manufacturing process to this factory, methyl alcohol could be replaced by ethyl alcohol which is less toxic and CNC machines need to be changed from open type to closed type. Second, government needs to provide more active administrative support. There needs to be an institutional system that employers who start a new business would be offered information including education of hazard materials, working environment measurement, regular checkups for employees, and other occupational safety and health measures. At the same time, especially for employers of small-sized or subcontracted factories, continuous education and systemic support for occupational safety and health should be implemented. Third, the institution for occupational safety and health should establish the system to communicate vulnerable workers about the occupational disease prevention directly. The current situation in which workers are excluded occupational safety and health system could replicate this weird tragedy that workers themselves do not recognize hazard of exposure materials. Establishing direct channel between workers and government as well as encouraging workers’ participation in workplaces was needed especially for small-sized enterprises.

## Conclusions

These cases occurred in a factory which manufactured vigorously smartphone parts hiring contract workers. The factory was a small-sized subcontracted firm of a major company and a blind spot to occupational health. Workers did not have appropriate ventilation system and proper personal protectors like gloves and masks against hazard materials. Working environment measurement and annual chekups for workers were not conducted. Increased demands for smartphones globally could lead the increase this kind of factory and more contract workers could be in risk to expose to hazard materials. For these reasons, our two cases have important meanings in view of occupational health. Contract workers in a small-sized subcontracted factory are placed more danger relatively than regular employees in a bigger factory. More attention is needed to protect their health.

## Abbreviations

AACT: American academy of clinical toxicology
ABGA: Arterial blood gas analysis
ACGIH: American conference of governmental industrial hygienists
BEI: Biological exposure indices
CNC: Computer numerical control
CT: Computed tomography
EEG: Electroencephalography
GC: Gas chromatography
ICU: Intensive care unit
KOSHA: Korea occupational safety and health agency
MMSE: Mini mental state examination
MRI: Magnetic resonance image
NCV: Nerve conduction velocity
OCT: Optical coherence tomography
STEL: Short term exposure limits
TWA: Time weighted average
